# Corridor-based relative severity assessment of the canine anaesthetic course

**DOI:** 10.3389/fvets.2026.1939921

**Published:** 2026-09-23

**Authors:** Julia D. Kschonek, Sabine B. R. Kästner, Philipp L. S. Ohland, Sara Lutscher, Holger A. Volk, Steven R. Talbot

**Affiliations:** 1 Department of Small Animal Medicine and Surgery, University of Veterinary Medicine Hannover, Hannover, Germany; 2 Department of Anaesthesia and Analgesia, University of Veterinary Medicine Hannover, Hannover, Germany; 3 Institute for Laboratory Animal Science, Hannover Medical School, Hannover, Germany; 4 Institute of Medical Statistics, RWTH Aachen University Hospital, Aachen, Germany

**Keywords:** anaesthesia, exploratory analysis, objective monitoring, RELSA, tasipimidine

## Abstract

The Relative Severity Assessment (RELSA) score grades welfare severity based on the magnitude of multivariate deviations from a reference value. Many variables monitored during anaesthesia are homeostatic and become harmful when they leave the normal range in either direction. Building on the corridor concept of the PVS from human intensive care, we conducted a feasibility study to assess the application and behaviour of a corridor extension of RELSA in two canine anaesthesia datasets. Each homeostatic variable was given a normal band, and only its deviation was folded into the standard RELSA algorithm. Severity in this report denotes the number this algorithm returns, and that number has not been shown to represent clinical severity. We obtained two Beagle datasets, including one four-arm within-subject study, allowing a paired between-protocol comparison. This is an exploratory secondary analysis of existing data, with no *a priori* power calculation. Under the selected bands, the calculated score changed across the recorded anaesthetic course: low while awake, rising at the documented propofol induction, maintained, and resolving on recovery. The dominant departures were hypotension, hypothermia, and mild hypercapnia, sometimes coinciding with a single animal, though their magnitudes are band-dependent. Corridor RELSA produced a number that changed across the recorded anaesthetic course and represented bidirectional departures as a single continuous score. The between-protocol comparison is not interpreted as evidence about the protocols because the verdict it returns depends on the variable panel being scored. The approach is a domain extension and a small refinement of the human-ICU corridor concept.

## 1 Introduction

Anaesthesia is a period of pronounced physiological derangement, and anaesthetic or sedation-related death remains a tangible risk in dogs, estimated at approximately 0.17% (approximately 1 in 600) in a large prospective survey (1). Monitoring generates many variables at once, each judged against its own reference range, and integrating them into a single reading of how deranged an animal is at a given moment is left to the anaesthetist. Current small animal guidelines organise that task by system, with separate recommendations for monitoring circulation, oxygenation, ventilation, body temperature, neuromuscular blockade, and anaesthetic depth (2). Combining these channels into a single continuous number is one way to make that reading explicit, and whether such a number adds anything to the individual measurements it is built from is a question that can be addressed directly in the data by comparing it with simpler summaries of the same out-of-band information.

The Relative Severity Assessment (RELSA) score grades welfare severity based on the magnitude of multivariate deviations from a reference value (3). Each variable is assigned a single direction of harm. By default, a fall below the reference value is penalised. For a variable in which a rise is harmful, the direction is “turned” so that the rise drives the score instead. The scheme is therefore monotonic, with one bad direction per variable. A variable that can be harmful in both directions therefore has to be handled in one of two ways under this scheme. Either it is assigned a single direction in advance so that departures on the other side leave the score unchanged, or it is left out of the panel altogether.

Many variables monitored during anaesthesia do not fit with this assumption. Blood pressure, body temperature, and blood gases are regulated within homeostatic ranges, and a departure in either direction can indicate physiological compromise. For these variables, a single adverse direction cannot capture clinically relevant changes. This two-sided case has already been addressed in human intensive care by the Patient Vital Status (PVS), which grades deviations relative to an age-normative corridor rather than a single threshold (4). The corridor extension is therefore a change in what the score can represent rather than a claim about how well it performs. Replacing the single reference value with a band allows a two-sided homeostatic variable to enter the score at all and allows a departure on either side of the band to register, without the analyst having to fix in advance which direction counts as harmful. Whether the resulting number is a more informative summary of the same out-of-band measurements than simpler alternatives is a separate question that the present study does not test.

Combining several monitored variables into one number is not new in itself. In human acute care, we aggregate weighted early warning scores sum contributions from routinely recorded vital signs into a single value that has been validated against subsequent deterioration and death (5). In veterinary medicine, the acute patient physiologic and laboratory evaluation score combines physiological and laboratory variables recorded within 24 h of admission into a diagnosis-independent index of mortality risk in hospitalised dogs (6). Within anaesthesia itself, derangement is more often quantified one variable at a time, as the duration for which a variable such as mean arterial pressure stays beyond a defined threshold (7). Each of those approaches is anchored to an outcome, which the present analysis is not. What we did not find reported is a corridor of this form applied to the monitored variables of an anaesthetic in a veterinary species and computed across the whole course rather than at a single admission time point.

The anaesthetic course also offers what continuous intensive-care data usually cannot: a scheduled event timeline against which a severity trace can be checked. Induction is documented to the minute, so an objective severity built from monitoring can be tested against a known event without being told where the event is.

We applied a corridor-based extension of RELSA to two canine anaesthesia datasets to examine its behaviour in this setting. Specifically, we examined whether the resulting severity score had changed across the documented anaesthetic course under the selected bands, identified the physiological variables that contributed most strongly to the score, and examined how far its behaviour depended on the choice of bands, variable panel, and aggregation rule. We did not set out to measure the effect of the anaesthetic-sparing protocols investigated in the original studies, and the four arms of the second dataset are used here to examine the stability of the score rather than to compare the protocols.

This study constitutes a retrospective, exploratory methods report on existing data. The score is used here in a descriptive role: at each recorded timepoint, it condenses the monitored measurements into a single number indicating how far they lie outside the bands assigned to them so that the number can be read against the documented course and traced back to the measurements that produced it. What is under test is therefore the behaviour of that calculation, namely whether it can be applied to routine and invasive canine anaesthesia monitoring, whether the number it returns changes across the recorded anaesthetic course under the selected bands, whether those changes are traceable to identifiable individual measurements, and how far all of this depends on the bands, the variable panel, and the aggregation rule. The level of evidence is correspondingly limited. The reference set is internal to these data; the bands are reference ranges rather than validated cut-points; and no external anchor, such as recovery quality, time to extubation, or a blinded severity rating, is available. No hypothesis was specified in advance, and no power calculation was performed. Nothing reported here establishes that the number measures clinical severity, that it adds information beyond simple summaries of the same out-of-band measurements, that it captures the effect of the anaesthetic protocol, or that it is ready for clinical or real-time use.

## 2 Methods

### 2.1 Datasets

We analysed two companion Beagle anaesthesia studies conducted by the same research group. Both were collected for other purposes, so the present study is an exploratory secondary analysis with no *a priori* sample-size calculation and is not powered to confirm small between-protocol differences. Study 1 is an anaesthetic-sparing (8) study of seven dogs, with 16 continuously monitored variables. In that study, isoflurane was titrated up and down in response to a nociceptive stimulus to determine the required isoflurane concentration under each of four premedication protocols, rather than held at a fixed maintenance level, and we did not further analyse this stimulus–response titration. Study 2 is a cardiovascular study (9) of seven dogs in a prospective, randomised, placebo-controlled, blinded, four-period within-subject (repeated-measures) design in which each dog received all four protocols in randomised order: an oral and intravenous placebo (PP), oral tasipimidine (TP), tasipimidine with methadone (TMP), and tasipimidine with methadone and dexmedetomidine (TMPD). Sampling spanned the course from awake baseline (M0) through induction and maintenance to recovery (M9). One dog was not anaesthetised under the TMP protocol for technical reasons, and no animal developed complications in this study.

### 2.2 Corridor extension and variable panel

Each homeostatic variable was assigned a normal band [lower, upper] from canine reference ranges or laboratory-Beagle distributions (mean plus or minus 2 SD), listed with the bands in Supplementary Table S1. These bands are reference values rather than validated cut-points, so conclusions are checked for robustness to band width and to band re-specification (Supplementary material). The excess outside the band, e = max(0, lower minus x, x minus upper), is zero for any value inside the band and grows with the distance from the nearer boundary. These excess values were incorporated into the standard RELSA algorithm, preserving the original procedure and replacing the single reference value with a reference corridor. The fold turns a two-sided homeostatic departure into the one-sided severity signal that RELSA expects. Throughout this paper, severity denotes the number the RELSA algorithm returns, that is, a unitless index of how far the recorded values lie outside their assigned bands, scaled against the reference set, and the term is retained only because it is the established name of that output. The index has not been shown to represent clinical severity. No outcome measure, welfare assessment, or blinded clinical rating was available in these datasets against which the number could be calibrated, so its correspondence to the clinical severity of an anaesthetic course is untested, and every value reported here should be read as a band-relative computation rather than as a graded clinical state. The only departure from PVS (4) is the band itself, an interval [lower, upper] with a zero-penalty interior in place of a single reference point.

The score itself is computed in four steps, which, together with the band table and the variable panel determine it completely. First, a band may be scaled by a factor k about its centre, so that for a centre c = (lower plus upper) divided by 2 and a half-width h = (upper minus lower) divided by 2, the scored band runs from c minus k times h to c plus k times h. The main analysis uses k = 1, and the bandwidth sweep reported in the Supplementary material varies k. Second, the excess is passed to RELSA as the pseudo-value of 100 minus e, so the deviation RELSA reads for that variable is e itself, and any value inside the band contributes a deviation of zero. All variables scored here are corridor variables, so the one-sided baseline-relative path of standard RELSA is available in the implementation but unused. Third, each deviation is divided by the largest deviation of the same variable in the reference set, yielding a relative weight between 0 and 1. The reference set is the scored data itself, so this denominator is internal to the dataset, and a variable that never leaves its band carries a weight of zero at every timepoint while still counting towards the denominator of the aggregation. Fourth, the severity at one timepoint is the root-mean-square of those weights, that is, the square root of the summed squared weights divided by the number of scored variables with a recorded value at that timepoint, and a variable without a value there enters neither the sum nor the count. The score therefore lies between 0 and 1 and is relative by construction. Every weight depends on the band table, the variable panel, and the composition of the reference set, so the score is defined only with respect to a stated band table and panel, and its sensitivity to those choices is itself one of the results reported here.

Study 2 retains the full invasive panel from the raw protocols, including pulmonary artery catheter haemodynamics and mixed venous blood gases, which is broader than the cardiovascular table reported in the source publication [Table 1, (9)]. We score the panel as recorded and note where individual variables are discarded, as they never leave the normal band.

### 2.3 Analysis

Severity was aggregated using the standard root-mean-square across scored variables, with depth (top three) and breadth (fraction of systems out of band) aggregations reported in the supplement for robustness checks. To ask what the aggregation adds over the measurements it is built from, we compared the corridor score with simpler summaries of the same out-of-band information, computed on the same phase-stable panel over the same 270 session-timepoints: breadth as defined above, the number of variables outside their band, the mean out-of-band excess across the panel, and, as a single-channel comparator, the out-of-band excess of invasive mean arterial pressure alone. Agreement with the corridor score was quantified by Spearman’s correlation, and the ability of each summary to separate awake from maintenance samples was quantified by the rank-biserial correlation. To locate the variance, we fitted a linear mixed model with random intercepts for timepoint, dog, and protocol (lme4), reading the four components as phase (timepoint), within-session (residual), dog, and protocol. To test whether severity differed across protocols, we used the within-subject (paired) unit of one summary per dog-protocol pair and a Friedman test blocked by dog, rather than pooling correlated within-session samples. That summary was the unweighted arithmetic mean of the corridor severity values over the six maintenance measurement points M3 to M8; each point contributed equally, and none was weighted by its duration. All six points carried a value in every session, and missing measurements were handled inside the score rather than at this stage, because the value at a measurement point is averaged over the variables actually recorded there, and a point is lost only when no scored variable was recorded at all. Because the measurement points are a protocol-defined ordered sequence rather than an elapsed-time axis, we did not compute an area under the curve. The test itself used complete blocks only; that is, the six of seven dogs with a session under all four protocols. The same blocked test, conducted during the awake window (M0 to M2), assessed whether tasipimidine premedication increased severity before induction. That comparison used the same unweighted mean, taken over the three awake measurement points. The band, the variable panel, and the aggregation rule are choices that define the score rather than settings that tune it, so their influence was analysed as a primary question and is reported in the Results and the Discussion rather than only in the supplement. The between-protocol test was accordingly repeated for each variable panel, with each session summarised by its mean, median, and maximum. Further analyses are documented in the Supplementary material: a between-protocol comparison within clinically pre-defined (but not registered, hence exploratory) subsystems and at each timepoint, both Benjamini-Hochberg corrected. A second removed the oxygen-tension variables, including their effect on the variance partition. A third swept the bandwidth and re-specified the bands to anaesthesia-appropriate ranges. Because not every channel is recorded at every phase, we also defined a fixed panel of the 16 variables present throughout, dropping respiratory rate, pulse oximetry, and end-tidal carbon dioxide, and recomputed the score from scratch on that panel with the reference set rebuilt within it. Two further panels were scored the same way: the fixed panel without the oxygen tensions (12 variables) and a reduced panel carrying one channel per physiological system (7 variables). For traceability, each score was decomposed by phase into the share of the total out-of-band weight that each variable carried. The reduced panel also addresses redundancy among the recorded channels: we assigned each scored variable to a single physiological system, counted how many channels each system contributed to the full panel, and quantified the agreement between near-duplicate channels using Spearman’s correlation. Finally, the between-protocol test was repeated with the median and with the maximum of the same maintenance window in place of the mean, which left it non-significant under all three rules (Friedman *p* = 0.284 for the mean, 0.308 for the median, and 0.457 for the maximum), so that comparison is far less sensitive to the choice of summary than it is to the choice of variable panel reported in the Results. Because only six complete blocks are available, the Friedman *p*-values were confirmed by within-block permutation. With that many blocks, the comparison can detect only large effects, so we report effect sizes (Kendall W) and per-protocol means alongside the *p*-values and do not interpret the comparison as evidence that the protocols are alike. Uncertainty in the variance components is reported as profile-likelihood confidence intervals, computed on the standard deviation scale so that they carry the units of the score itself. The phase (timepoint) component is 0.100 (95% CI 0.064–0.160), the session within the dog is 0.035 (0.019–0.047), and the residual is 0.065 (0.059–0.071), while the dog component is 0.007 with an interval of 0.000–0.038 that reaches the boundary, so it is read as an upper bound rather than an estimate. Analyses were run in R (v4.5.3) with the RELSA and lme4 packages. All statistical analyses were designed and implemented by an author based at the Institute of Medical Statistics, RWTH Aachen University Hospital (S.R.T.), who is responsible for the analysis code (see Author contributions).

Because the design is hierarchical, with repeated timepoints within a session and several sessions per dog, the variance model explicitly includes the session level. A session, meaning one dog under one protocol, was entered as a random intercept nested within each dog so that repeated timepoints from the same session are not treated as independent, and the timepoint remained random as the phase component. Serial dependence was then estimated directly by refitting the same model in nlme with a first-order autoregressive, AR(1), residual correlation over timepoint within session, and the two error structures were compared by AIC. Both partitions, before and after this respecification, are reported to make the effect of carrying at the session level visible.

The model was fitted in lme4 syntax as relsa ~ protocol + (1 | dog/session) + (1 | timepoint). Here, relsa is the root-mean-square corridor score for a single session-timepoint; protocol is the four-level treatment arm; session is the dog-protocol pair nested within dog; and timepoint is the M0 to M9 sampling index, entered as an unordered factor. Variance components were estimated using restricted maximum likelihood and are reported as the share of the total random effects and residual variance attributable to each term.

## 3 Results

### 3.1 The calculated score changed across the recorded anaesthetic course under the selected bands

In Study 2, the score calculated under the bands listed in Supplementary Table S1 was low while the dog was awake, rose at the documented propofol induction (median time point M3 in 26 of 27 sessions), remained elevated during maintenance, and fell during recovery (Figure 1A). The number of variables contributing to each value increased from approximately 14 awake to approximately 16–17 at maintenance as the invasive channels came online (Figure 1B), so the panel that feeds the score is not the same at every phase, and the rise could in principle follow from that change in what is recorded rather than from the recorded values. We therefore repeated the analysis on a panel held fixed across phases, reported next. In the respecified variance partition, the protocol entered as a fixed effect because four levels are too few to support a variance component. The timepoint (phase) component accounted for 65% of score variance, the within-session residual for 27%, session-level clustering for 8%, and dog for under 1%. Added protocol did not improve the fit over the intercept-only model (likelihood-ratio chi-square = 4.82, *df* = 3, *p* = 0.185), and the three tasipimidine protocol contrasts lay within 0.05 score units of the placebo protocol. The protocol is, therefore, reported as a fitted contrast on this variable panel rather than as a share of variance, and it is not read as evidence about the protocols themselves. This phase component is inflated by the oxygen-tension variables, which carry room-air bands and so breaches only during maintenance: with them removed, the phase share falls to approximately 20%, and the within-session residual dominates (Supplementary material). The defensible reading is therefore narrow. Under these bands and the variables recorded in these sessions, the calculated score rose at the documented induction, a timing not supplied to the score, and fell again on recovery. That is a description of the recorded course under one band specification rather than a demonstration that the score tracks anaesthesia in a general sense, and the share of variance it explains depends on which variables carry the bands rather than on the score itself. This temporal rise would also appear under one-sided RELSA. The corridor’s distinct contribution is confined to bidirectional departures described next.

**Figure 1:**
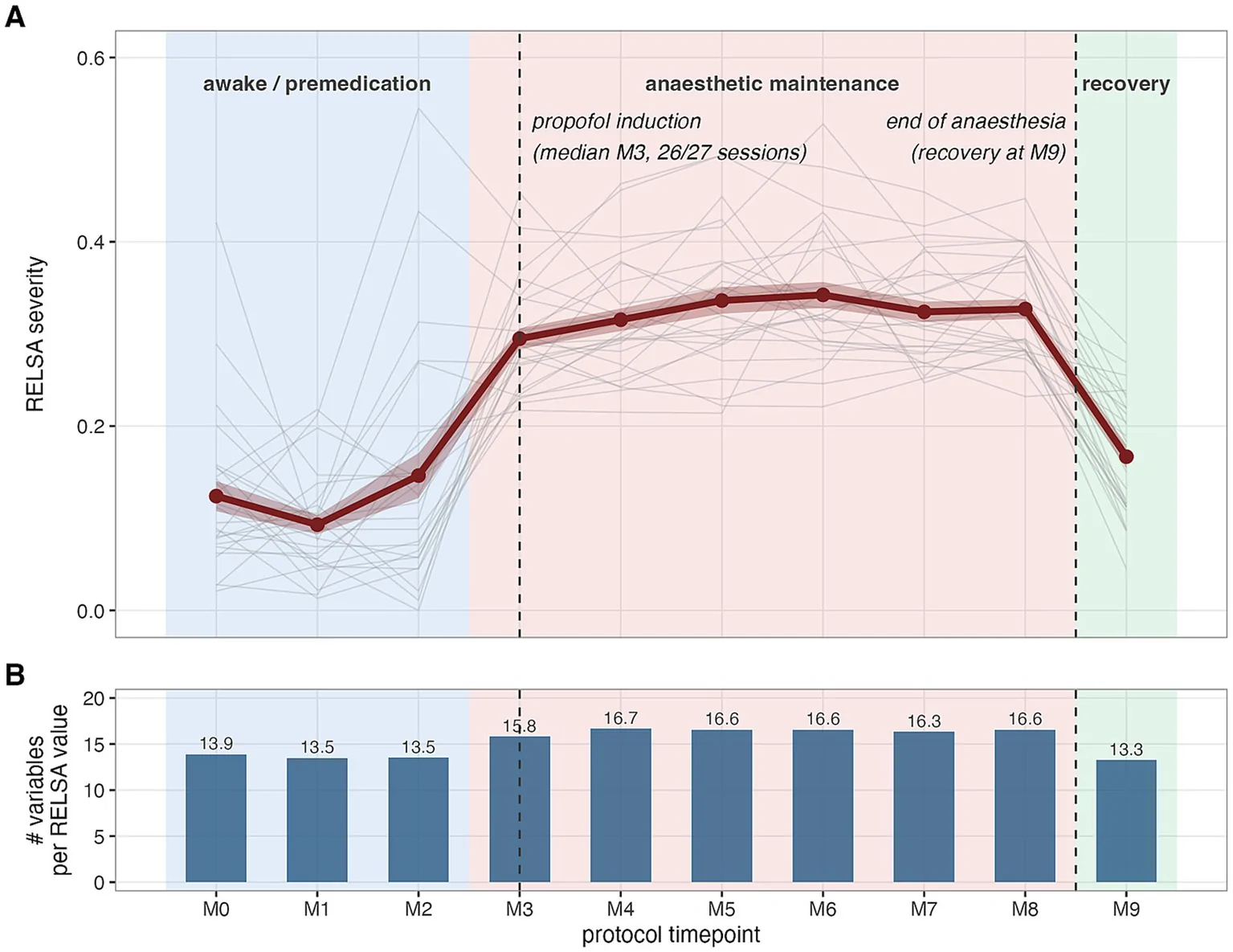
Corridor RELSA across Study 2 (27 sessions). Sessions come from seven dogs, and each value is the standard root-mean-square aggregate across the variables recorded at that timepoint, using the bands listed in Supplementary Table S1. **(A)** Severity is minimarameter name pathsthesia (recovery at M9). The bold line is the session mean with standard error, and the faint lines are individual sessions. **(B)** The number of variables averaged into each value rises from awake to maintenance as the invasive channels come online. Panel composition therefore changes with the anaesthetic phase, from approximately 14 variables awake to approximately 16–17 at maintenance, so the trace in **(A)** is not computed from a constant variable set.

Carrying the session level explicitly moves part of what the first model had left in the within-session residual. Sessions, meaning one dog under one protocol, took approximately 8% of the score variance; the residual fell from 32% to approximately 27%, and the dog component fell to under 1%, while the phase component was essentially unchanged at approximately 65%. Successive timepoints within a session were also correlated, with an estimated AR(1) coefficient of 0.60 between adjacent timepoints, and the AR(1) error structure fitted the recorded scores better than independent errors (AIC -438.1 against -342.0). Repeated samples from the same session therefore carry less independent information than their number suggests, so an analysis that pooled them as independent observations would overstate its precision.

Three of the 19 scored channels are recorded almost only under anaesthesia (respiratory rate, pulse oximetry, and end-tidal carbon dioxide, present in 0–14% of awake rows against 95–97% of maintenance rows), while the remaining 16 are present in 89–100% of rows in every phase. Recomputing the score from scratch on that fixed 16-variable panel, with the reference set rebuilt within it, leaves the course intact: mean score 0.122 awake, 0.346 during maintenance, and 0.168 on recovery, against 0.121, 0.323, and 0.167 on the full panel, with the maintenance mean above the awake mean in 27 of 27 sessions and the peak inside the anaesthetised window M3 to M9 in 26 of 27 (rank-biserial awake against maintenance 0.91, against 0.90 on the full panel). The change in the number is also traceable to identifiable measurements. On the fixed panel the three largest contributors carry 54% of the total out-of-band weight while the dog is awake (core temperature 21%, invasive systolic pressure 18%, central venous pressure 15%), 61% during maintenance (PaO2 29%, SvO2 17%, oscillometric mean arterial pressure 16%), and 57% during recovery (core temperature 28%, oscillometric mean arterial pressure 19%, central venous pressure 10%). The size of the step is nevertheless a property of the panel and not of the sessions alone. On a 12-variable fixed panel without the oxygen tensions, the awake-to-maintenance step falls from +0.225 to +0.120, and the phase share of the variance is from 68 to 26%, with the maintenance mean still above the awake mean in 25 of 27 sessions, and on a 7-variable panel carrying one channel per physiological system, the step rises to +0.308 (Supplementary material).

### 3.2 Bidirectional departures captured by the corridor

During maintenance of anaesthesia in Study 2, the largest departures from the awake reference band were hypotension and hypothermia on the low side and mild hypercapnia on the high side (Figure 2A). Invasive mean arterial pressure fell below its band in 79% of maintenance samples (median 69 mmHg), and oscillometric pressure in 94% (median 61 mmHg), and core temperature fell below its band in 83% of samples (median 37.6 °C). These are mild, expected anaesthetic perturbations rather than frank morbidity, and much of their recorded magnitude reflects an awake band applied under anaesthesia, as we discuss further below.

**Figure 2:**
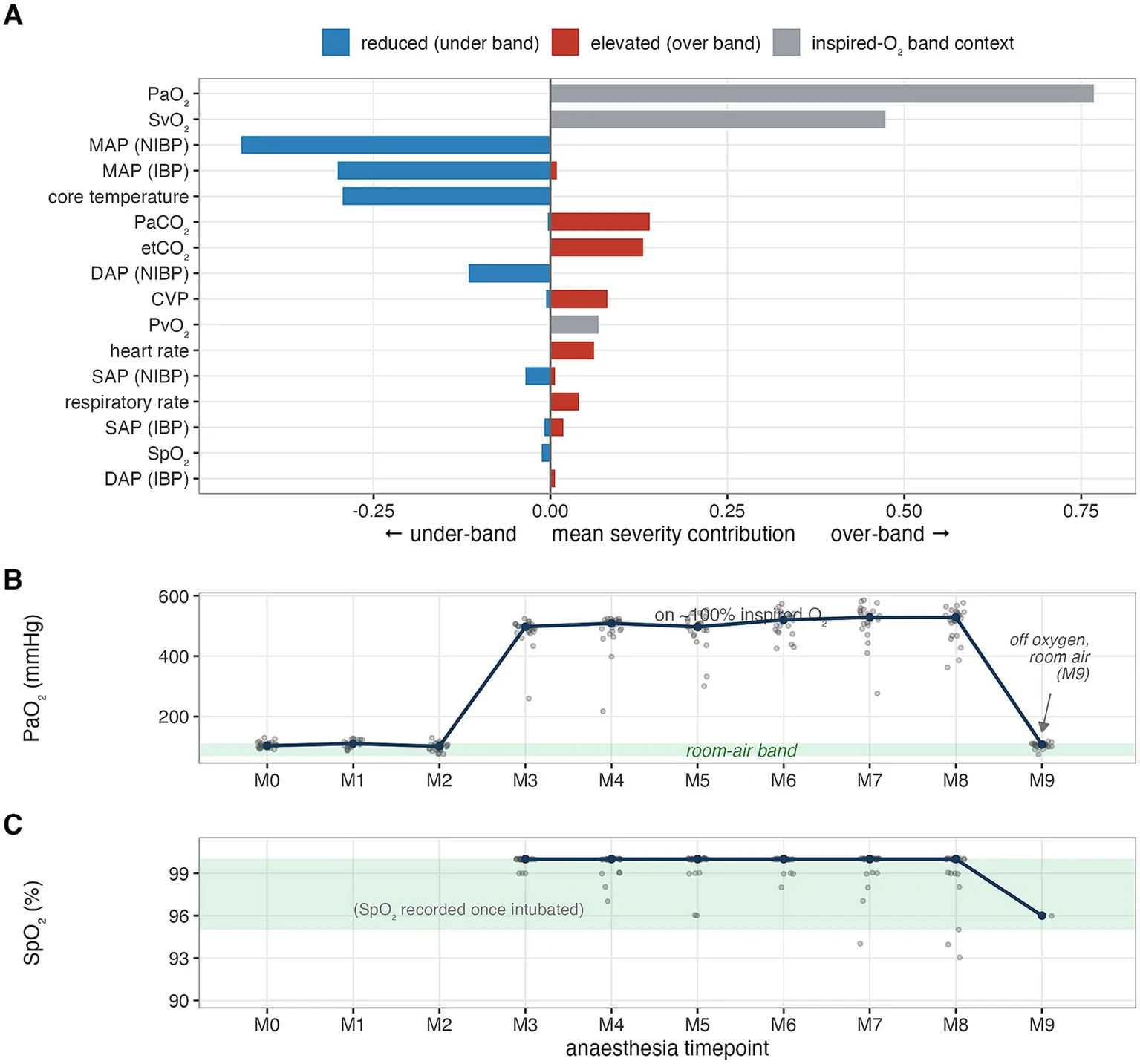
Direction-resolved drivers, Study 2 maintenance phase. Panel **(A)** uses the maintenance timepoints M3 to M8 of the 27 sessions, while **(B)** and **(C)** run across the whole recorded course from M0 to M9. **(A)** Each variable's mean severity contribution is split into under-band (blue, too low) and over-band (red, too high). The sign is a plotting convention that separates the two directions, and a departure on either side adds to the score. Hypotension (MAP) and hypothermia (core temperature) dominate the low side, and mild hypercapnia (PaCO_2_, etCO_2_) the high side. PaO_2_ and SvO_2_ (grey) exceed their room-air bands under therapeutic oxygen and are a band-specification artefact, not a derangement. **(B)** PaO_2_ swings between room air and oxygen across the course, dropping back to the room-air band at M9 once the dog is off oxygen on recovery, while **(C)** SpO_2_ stays at the ceiling, so a saturation-only view cannot resolve the oxygen tension. In **(B,C)** the shaded band is the reference band, the grey points are the individual measurements, and the line joins the timepoint medians.

Arterial carbon dioxide sat at the upper edge of its band overall (median 45.5 mmHg, above the band in approximately half of the samples). It rose higher in the methadone-containing protocols (TMP and TMPD), with end-tidal carbon dioxide following the same pattern. The high-side carbon dioxide breach, present in approximately half of maintenance samples, is a direction that a one-sided decrease-is-bad score cannot capture, and, in a given animal, it coincides with low-side hypotension and hypothermia. The size of this co-occurrence is band-dependent, falling sharply under anaesthesia-tolerant bands (Supplementary material), yet the corridor contributes to the two-sided departure.

Heart rate fell with the alpha-2 agonist but remained within the wide reference band (50–118 beats per minute), so it contributed almost nothing to severity, indicating that the corridor scores out-of-band excursions rather than the direction of a within-range change.

Arterial oxygen tension (PaO_2_) sat far above its band during maintenance, the expected consequence of near-pure inspired oxygen rather than a derangement. PaO_2_ was bimodal by phase, near 105 mmHg while the dog breathed room air (awake and recovery) and near 509 mmHg while the dog breathed oxygen (maintenance). The inspired fraction was not recorded in Study 2, so it is inferred from the documented near-100% oxygen and the companion study, against which the resulting maintenance P/F ratio is close to expected.

Mixed-venous oxygen saturation (SvO_2_) was likewise elevated, reflecting, under anaesthesia, reduced oxygen consumption, together with a larger dissolved-oxygen contribution to venous content under hyperoxia rather than inspired oxygen as such. The elevated mixed-venous oxygen tension (PvO_2_) is consistent with this. Pulse oximetry read 99–100% throughout and could not resolve the difference (Figures 2B,C). We therefore treat the PaO_2_ excursion as a band-specification issue rather than as pathology and note that bands for inspired-oxygen-dependent variables must be indexed to the inspired fraction. In contrast, mixed-venous variables need anaesthesia-state re-specification.

### 3.3 The between-protocol verdict depends on the variable panel

Study 2 contains four treatment protocols, and we treat them here as a feature of the source dataset rather than as a comparison so this score can settle. The arms are retained for one purpose: to show that the between-group verdict that the scores return is not stable. Scoring the same maintenance sessions under four defensible variable panels, with the score recomputed from scratch and its reference set rebuilt inside each panel, reverses the answer. The blocked Friedman test over one mean per dog and protocol gives *p* = 0.284 on the full 19-variable panel, *p* = 0.024 on the 16-variable phase-stable panel that drops the three channels that come online only under anaesthesia, *p* = 0.334 on the 12-variable panel that also removes the oxygen-tension channels, and *p* = 0.012 on a 7-variable panel carrying one channel per physiological system (Figure 3E). Within-block permutation reproduces each value (*p* = 0.325, 0.021, 0.372, and 0.005). The measurements are identical in all four cases, and the panels differ only in which of the mutually correlated channels enter the aggregate, so the reversal is a property of the scoring choice rather than of the animals. The awake premedication window is shown on the same basis in Figure 3D (Friedman *p* = 0.20) and carries the same dependence. We therefore report the panel dependence as a result and draw no conclusion about the protocols from the score.

**Figure 3:**
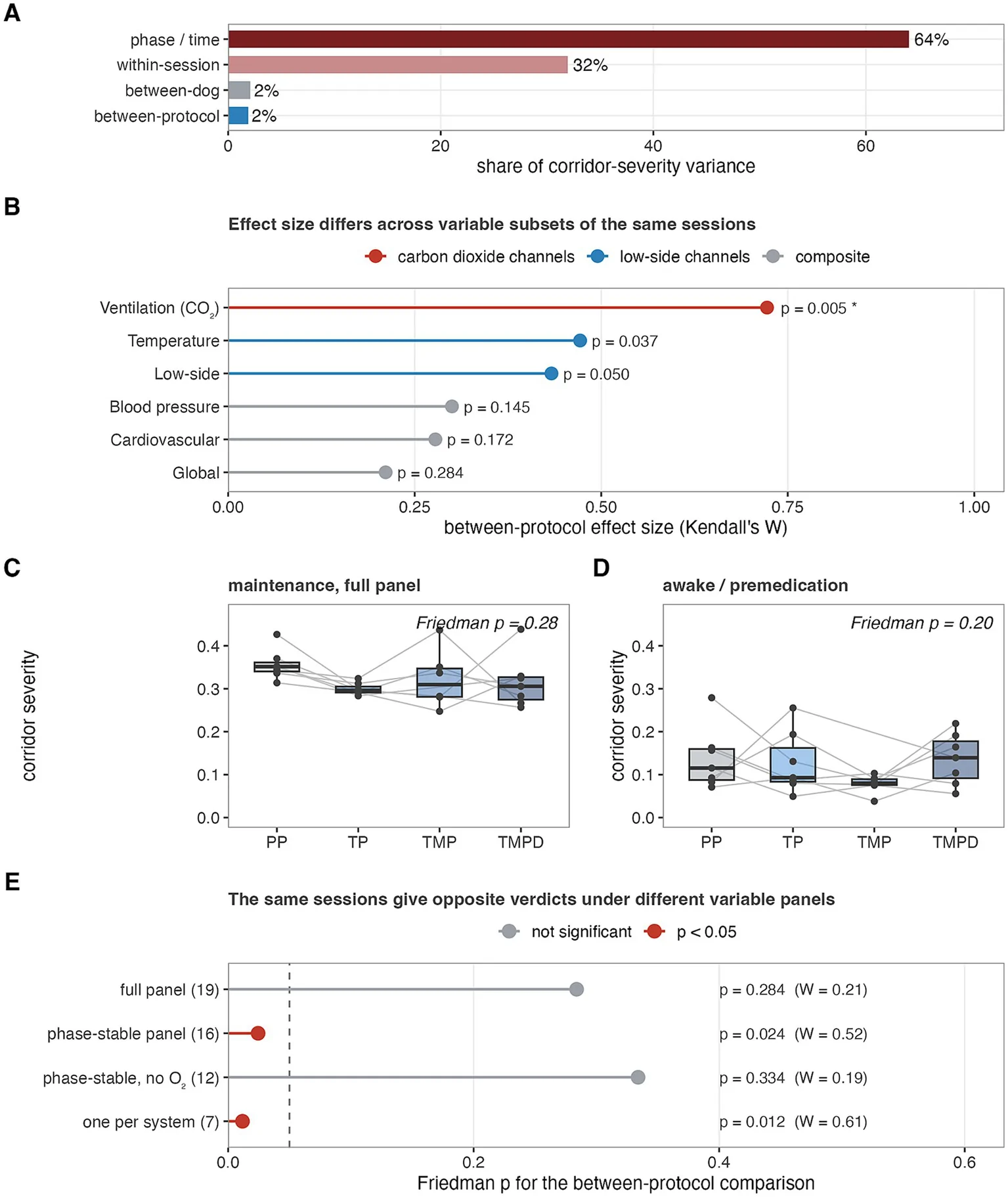
Panel dependence of the between-protocol comparison in the Study 2 within-subject design. (**A**) Variance partition, in which the anaesthetic phase dominates, and the treatment protocol accounts for about 2%. **(B)** Between-protocol effect size (Kendall *W*) and Friedman *p* for the corridor severity of each clinical subsystem, showing that the effect size differs across variable subsets of the same sessions. The ventilation subsystem (arterial and end-tidal carbon dioxide) is the only subset to cross the corrected threshold (red), and the temperature and low-side subsets do not (blue). **(C)** Maintenance severity by protocol on the full variable panel. Each point is one dog-protocol mean over M3 to M8, and the blocked Friedman test on those values returns *p* = 0.28. **(D)** The same comparison in the awake premedication window, which returns *p* = 0.20. **(E)** Friedman p for the between-protocol comparison recomputed under four defensible variable panels: the full 19-variable panel (*p* = 0.284), the 16-variable phase-stable panel (*p* = 0.024), the 12-variable phase-stable panel without the oxygen tensions (*p* = 0.334), and the 7-variable one-channel-per-system panel (*p* = 0.012). The same sessions therefore give opposite verdicts under different variable panels, so panels **B** to **E** are shown as a property of the score rather than as evidence about the protocols. No equivalence margin was set and no equivalence test was run, and the figure is not evidence that the protocols are alike. Grey lines link the same dog across protocols. Protocols are PP (placebo), TP (tasipimidine), TMP (tasipimidine plus methadone), and TMPD (tasipimidine plus methadone plus dexmedetomidine).

The subsystem analysis is a further instance of the same dependence. A two-variable ventilation panel of arterial and end-tidal carbon dioxide separates the arms (Friedman *p* = 0.005, *q* = 0.03, Kendall *W* = 0.72, within-block permutation *p* = 0.0008; Figure 3B), while the full panel of the same sessions does not, so the effect size attached to a protocol depends on which subset of the recorded channels is scored. Arterial carbon dioxide itself was near 51–52 mmHg in the protocols containing methadone, compared with approximately 40 mmHg in the others. That difference sits in the raw measurements and does not depend on any band, but we do not read it as a protocol effect here, because the ventilation mode and any intra-anaesthetic management of carbon dioxide were not recorded in the source data. Scored as corridor severity, the same difference weakens under a permissive anaesthesia carbon-dioxide band and is then marginal (Supplementary material).

Descriptively, the placebo arm shows greater hypothermia, while the mean arterial pressure severity is flat across the arms. We report these as features of the recorded dataset and no longer offer them as a balance of opposing protocol effects, because the aggregate that would carry such a reading is the same aggregate whose verdict moves with the panel. The same dependence appears in the other two analytical choices. The aggregation rule moves the same test, the full-panel p being 0.284 with each session summarised by its mean, 0.308 by its median, and 0.457 by its maximum, and widening the bands to anaesthesia-tolerant values shrinks the severity magnitudes themselves (Supplementary material). The four arms therefore appear here as a demonstration that the score’s between-group verdict depends on how the score is configured rather than as evidence about the protocols.

The recorded panel is not balanced across physiological systems. Perfusion pressure contributed six of the 19 channels (invasive and oscillometric systolic, mean, and diastolic pressure), oxygenation three (PaO2, SaO2 and SpO2), filling pressure three (central venous, pulmonary arterial, and wedge pressure), ventilation two (PaCO2 and end-tidal carbon dioxide), and venous oxygen two (SvO2 and PvO2), while thermoregulation, cardiac rate, and respiratory rate contributed one channel each. The near-duplicate channels agreed closely, with Spearman correlations of 0.94 between PaO2 and SaO2, 0.95 between SvO2 and PvO2, 0.77 between PaCO2 and end-tidal carbon dioxide, and 0.76, 0.65, and 0.72 between the invasive and oscillometric mean, systolic, and diastolic pressures. The weight a system carries in the aggregate therefore depends in part on how many channels happen to be recorded for it. Recomputing the score on a reduced panel of one channel per system (invasive mean arterial pressure, PaCO2, core temperature, heart rate, PaO2, SvO2, and central venous pressure) reproduced the same recorded course, with means of 0.127 awake, 0.435 during maintenance, and 0.179 on recovery, maintenance above awake in 27 of 27 sessions, and the peak inside the anaesthetised window in 27 of 27, and the reduced-panel and full-panel scores tracked one another closely across all timepoints (Spearman rho = 0.93). The between-protocol comparison did not survive the same reduction. It was null on the full 19-variable panel (Friedman *p* = 0.284) and nominally significant on the seven-variable one-channel-per-system panel (Friedman *p* = 0.012, within-block permutation *p* = 0.005, Kendall *W* = 0.61), so the between-group verdict on these sessions is set by the choice of panel rather than by the sessions themselves (Figure 3E).

### 3.4 Cross-study reproduction of the low-side pattern

The low-side signature of hypotension with hypothermia was reproduced in independent Study 1. Study 1 also recorded an inspired oxygen level near 97%, providing context for the high arterial oxygen tension observed in Study 2. In neither study did severity increase with anaesthetic dose, whether propofol in Study 2 or isoflurane depth in Study 1. We do not read this as evidence about the protocols or about anaesthetic dose, because the between-group verdict of the score is not stable across defensible variable panels (Figure 3E). These cross-study points are provided in the supplement, while the sensitivity of the score to the bands, the variable panel, and the aggregation rule is carried in the main text, because it is a property of the measurement rather than a technical check on it.

The score tracks simpler summaries of the same out-of-band measurements. Across all 270 session-timepoints, the corridor score correlated with the mean out-of-band excess at Spearman rho 0.90, with breadth at 0.80, with the number of variables out of band at 0.75, and with the out-of-band excess of invasive mean arterial pressure alone at 0.66. It also separated the awake from the maintenance samples no better than those summaries did, with rank-biserial separations of 0.91 for the corridor score, 1.00 for the mean out-of-band excess, 0.84 for the count, 0.80 for breadth, and 0.61 for pressure alone. These data therefore provide no evidence that the aggregation carries information beyond the individual out-of-band measurements it is built from, and are reported here as one possible way of summarising them rather than as a necessary or superior one.

## 4 Discussion

Corridor RELSA produced a single continuous trace that changed across the recorded anaesthetic course under the selected bands, rising at documented induction and resolving on recovery, with contributions from departures from homeostasis in both directions. Replacing the single reference value with a reference corridor allows blood pressure, temperature, and blood gases to enter the score without an imposed direction of harm. The score could be computed at every recorded timepoint using the measurements already included in the protocols. Whether it measures physiological burden, whether it could support the refinement of anaesthetic protocols, whether it carries information about outcomes, and whether it could be used in real time are hypotheses that this retrospective analysis cannot test, and we present them as hypotheses below. Testing them would require a prospective study with an external anchor, such as recovery quality, time to extubation, or a blinded severity rating, together with signalment-specific reference corridors that account for breed, age, and other relevant canine characteristics.

What the two-sided fold changes are is that departures can enter the number at all. Hypotension and hypothermia run with mild hypercapnia in the same animal, and a one-sided index that fixed a single adverse direction per variable would not register the high-side departure. How often the two sides coincide is itself band-dependent and falls sharply under anaesthesia-tolerant bands, so this is a property of the calculation under the bands we selected rather than evidence that the two-sided number grades these animals more correctly. The heart-rate results make a complementary point.

Tasipimidine, a selective alpha-2A agonist (10), lowered heart rate from approximately 113 beats per minute in the placebo protocol to approximately 68 in the protocol that added dexmedetomidine (across protocols, omnibus p approximately 0.002), a large and pharmacologically expected effect that, however, produced no corridor severity because the rate stayed inside the 50–118 beats per minute band. A value of 68 beats per minute therefore contributed little to the calculated score; this does not establish that it was physiologically normal or clinically benign. For the calculation, the selected band, rather than the direction of a change within that band, determines whether heart rate contributes to the score. At the same time, the score could not fully represent the cardiovascular effects of alpha-2 agonism because cardiac output lacked an agreed-upon band and was excluded.

The oxygen variables are a caution rather than a finding. Arterial oxygen tension exceeded its room-air band throughout maintenance. Still, it was close to what near-pure inspired oxygen predicted, and the corridor flagged it only because a room-air band was applied while the patient was on therapeutic oxygen. The practical lesson is that bands for inspired oxygen-dependent variables should be indexed to the inspired fraction, and that mixed-venous variables need an anaesthesia state rather than an inspired-fraction re-specification. We report these excursions as a band-specification issue rather than as hyperoxic pathology. Hyperoxia can become clinically relevant during prolonged anaesthesia or in intensive care (11). Still, at the approximately 2-h anaesthetic duration of this study, we did not treat the elevated oxygen tension as pathological exposure.

Because Study 2 is a within-subject study, the between-protocol comparison rested on six complete dog-by-protocol blocks (five for arterial carbon dioxide and invasive pressure, reflecting missing samples) from seven dogs. We nevertheless make no claims about the protocols on this basis, and three obstacles stand in the way that a larger sample would not be removed. The washout between periods is not recorded, so a carryover effect cannot be excluded. The intra-anaesthetic management of each session, including the ventilation mode, fluids, and any cardiovascular support, was not recorded either, so a between-group difference in the score could not be attributed to the protocol rather than to how that individual anaesthetic was run. Above all, no expected direction or magnitude of score change was specified before the analysis, and the verdict itself moves from *p* = 0.284 to *p* = 0.012 with the choice of variable panel, so the comparison cannot carry a protocol-level interpretation in either direction. The anaesthetic-sparing effect remains the result reported by Kästner et al. (8), and the present analysis neither adds to it nor qualifies it. What the four arms contribute here is a stress test of the score, and the instability they expose is one of this report’s findings.

Several boundaries apply. The word severity is used here in its RELSA sense and carries no validated clinical meaning in this setting, because nothing in either dataset ties the computed number to a clinical judgement of how the anaesthetic course affected the dog, so no clinical inference should be drawn from its magnitude. The corridor concept is PVS (4), so this study is a domain extension and a small refinement: an interval band with a zero-penalty interior in place of a point reference, rather than a new method. The reference set is data-driven, so every comparison is internal, and severity magnitudes are not comparable across studies. The reference bands come from laboratory and dog ranges and clinical ranges. They are not validated anaesthesia cut-points, and the inspired-oxygen-dependent bands were mis-specified under therapeutic oxygen, as discussed. What counts as normal also varies with breed and age, so the bands are population-specific. Sighthounds differ from other breeds in resting haematocrit and arterial pressure, and paediatric or geriatric animals differ again, so applying the corridor beyond adult laboratory Beagles would need breed- and age-appropriate bands. The fold also treats a departure below the band and one above it as equally harmful for a given distance.

In contrast, the two directions can carry different clinical consequences, for example, hypotension against hypertension or hypothermia against hyperthermia. Direction-specific bandwidths or weights would allow the score to carry this asymmetry and would be a natural extension. The scored variables are not physiologically independent either, so co-occurring breaches such as hypotension, hypothermia, and hypercapnia partly reflect shared mechanisms rather than separate insults, and an aggregate that sums correlated channels can register overlapping information more than once. This is quantified here rather than assumed. Perfusion pressure entered the full panel through six channels, and oxygenation and filling pressure through three each, whereas thermoregulation and cardiac rate entered through one each, so the aggregate weights a system partly by how densely it was instrumented. Reducing the panel to one channel per system left the recorded course intact but reversed the between-group verdict, which is why the variable panel is reported here as a determinant of the result rather than as a fixed property of the method. The dependence of the number on these choices is one of the findings of this study rather than one of its boundaries. Because the bands are wide ranges applied under anaesthesia, the low-side severity, the size of the induction arc, and the two-sided co-occurrence shrink markedly when the bands are widened to anaesthesia-tolerant values (Supplementary material). The size of the arc changes again with the variable panel, and the between-group verdict reverses with it: it is null on the full panel and on the phase-stable panel without the oxygen channels, while reaching nominal significance on the phase-stable and one-channel-per-system panels. What holds across these configurations is the direction of the within-session change rather than any magnitude. Every magnitude reported here, and any between-group reading of it, is a property of the chosen band, panel, and aggregation rule as much as of the animals, so a corridor score is interpretable only when those three choices are stated with it. The convergent validity against acidosis is partly circular because arterial carbon dioxide is itself a measured variable, and the genuinely metabolic residual is weak (a partial correlation of approximately −0.20). The sample comprises seven dogs, and no power analysis was performed, although the repeated-measures design provides paired power for the within-subject contrasts. An external outcome anchor, such as recovery quality, time to extubation, or a blinded severity rating, is absent here, but it is not the single remaining step. The specification of the score is itself unsettled. The bands are reference ranges rather than anaesthesia cut-points, the panel of variables available to the score changes with the phase of the anaesthetic, and the root-mean-square is one of several defensible ways of summarising the same out-of-band measurements. These choices are not neutral. In the same sessions, the between-protocol verdict reverses with the variable panel (Figure 3E), and what the corridor aggregate adds over simpler summaries of the same out-of-band information is not established here. An outcome anchor cannot adjudicate between versions of a score that can still be respecified once the anchor is known, so fixing the panel, justifying the bands against the anaesthetised state, and showing what the aggregation adds are the prior steps, and outcome anchoring follows them.

What these datasets can and cannot establish is worth stating in one place. They demonstrate that the corridor calculation could be computed from routine and invasive canine anaesthesia monitoring data, that the number it returns changed across the recorded anaesthetic course under the bands used here, that those changes can be traced back to identifiable individual measurements, and that the number is sensitive to the choice of bands, to the variable panel, and to the aggregation rule, a sensitivity that is itself one of the results reported here. Several properties remain untested. Neither dataset ties the number to a clinical judgement of how the anaesthetic course affected the animal, so it is not shown to measure clinical severity. It tracks simpler summaries of the same out-of-band measurements closely, and these data do not show that it adds information beyond them. Its between-group verdict changes with the variable panel (Figure 3E), so these data do not support reading it as a measure of protocol effects. No external outcome anchor was available in either dataset, so nothing here bears on prediction, and neither real-time nor clinical use was tested. Those questions belong to a prospective study designed to answer them.

Real-time use was not tested here, so what follows sets out how such a test might be designed rather than how a monitor should be run at the patient. The variables that carried the signal here are routinely and non-invasively available, so a prospective evaluation could be built on a minimal panel of mean arterial pressure, core temperature, end-tidal carbon dioxide, heart rate, and oxygen saturation, with the invasive pressures and mixed-venous gases added only when an arterial or pulmonary-artery line is in place. Such a panel cannot be assumed to inherit the behaviour reported here, because the panels we examined did not behave alike, so it would have to be evaluated in its own right. Two design choices matter more than the exact list. First, the bands should match the anaesthetised state rather than the awake animal, since awake bands allow the expected anaesthetic hypotension, mild hypothermia, and hyperoxia to be registered as severity. Second, oxygen saturation is better scored as a one-sided floor than as a corridor, because it sits at its ceiling under therapeutic oxygen. A single number does not replace per-system monitoring, which is what drives an intervention. Whether the number is useful alongside the individual channels is the hypothesis such a study would have to test, and it would have to be reported with its depth (the top contributors) and breadth (the fraction of systems out of band) so that the deranged system stays visible. A candidate minimal panel for prospective validation, with suggested anaesthesia-appropriate bands and the rationale for including or dropping each variable, including those that contributed nothing here, is given in Supplementary Table S6. That list is descriptive rather than recommended. It records which channels carried out-of-band weight in seven laboratory Beagles from a single research group; it was not tested against any outcome, and its composition is band-dependent, since the largest maintenance contributor in these data was arterial oxygen tension scored against a room-air band, which we read as a mis-specification rather than as a finding. Two of its channels, end-tidal carbon dioxide and oxygen saturation, were also recorded only after induction here, so the panel could not be scored across the whole anaesthetic course in this dataset. Prospective testing against an external anchor would be needed before any such panel is put forward for clinical monitoring.

Whether a continuously updated severity score that integrates multimodal monitoring into a single number gives a more objective and comparable reading of physiological burden during anaesthesia is a hypothesis this study sets up rather than one it answers. The present results indicate that the corridor extension was computationally feasible in these datasets and that, under the selected bands, the resulting score changed across the documented course reported in the source studies. What comes first is a fixed variable panel, bands justified for the anaesthetised state, and evidence of what the aggregation adds over simpler summaries of the same measurements. Validation against an external anchor, in larger datasets and in other species, follows those steps rather than replacing them.

## Data Availability

The data analysed in this study is subject to the following licenses/restrictions: The data are not publicly available due to ethical and privacy restrictions, i.e., any data transfer to interested persons is not permitted without a specific formal contract. This contract is available to qualified researchers and includes a guarantee of compliance with the obligation to maintain data confidentiality in accordance with the provisions of the German data protection law. Interested cooperative partners who can sign a contract (between authors, as well as between members of the University of Veterinary Medicine Hannover and members of the funding institution) may contact the corresponding author. Requests to access these datasets should be directed to corresponding author (julia.dorothee.kschonek@tiho-hannover.de).
