# Supplementary material for: Corridor-based relative severity assessment of the canine anaesthetic course
Source: Front Vet Sci. 2026 Sep 23;13:1939921. doi: 10.3389/fvets.2026.1939921 (PMC13643907; doi:10.3389/fvets.2026.1939921)
Supplement: Supplementary file 1 [file Supplementary_file_1.DOCX]

**Supplementary Material**

**Supplementary Methods**

**S1. The corridor fold**

Each homeostatic variable is assigned a normal band [lower, upper]. The excess outside the band, e = max(0, lower − x, x − upper), is zero inside the band and grows with distance from the nearer boundary. The pseudo-value handed to the standard RELSA algorithm is 100 − e, so a corridor variable is scored like a one-sided decrease-is-bad variable while remaining penalised in both directions. One-sided welfare variables are left baseline-relative through the standard normalisation, and excluded variables are dropped. A single scaling knob k widens or narrows every band about its centre for sensitivity analysis (k = 1 in the main results). The fold is a pre-transform and changes nothing inside the RELSA package.

**S2. Pre-specified subsystem panels**

To ask whether the full-panel between-protocol result hides a difference in one physiological system, severity was recomputed on pre-specified subsets of the corridor variables and compared between protocols. The panels were Global (all corridor variables), Cardiovascular (all pressures, heart rate, central venous, pulmonary, and wedge pressure), Blood pressure (systolic, mean, and diastolic by both methods), Ventilation (arterial and end-tidal carbon dioxide), Temperature (core temperature), and a Low-side panel (invasive and non-invasive mean arterial pressure and core temperature). Each was tested with a Friedman test blocked by dog on the per-dog-per-protocol maintenance mean, with Benjamini-Hochberg correction across the panels and Kendall’s W as the effect size. The panels overlap (Global spans every variable, Temperature is a subset of the Low-side panel), so the subsystem analysis is exploratory; excluding the Global umbrella from the correction leaves the ventilation subsystem the only one below q = 0.05 (q = 0.025). As reported in the main text, these subsystem verdicts are themselves an instance of the dependence of the between-protocol comparison on the variable panel, and no conclusion about the protocols is drawn from them.

**S3. Multiplicity, per-timepoint tests, and effect sizes**

Three multiplicity layers were controlled. Across the subsystem panels, the six p-values were Benjamini-Hochberg corrected. Across the ten timepoints, M0 to M9, the per-timepoint Friedman p-values were Benjamini-Hochberg corrected. In the awake window, the placebo-versus-arm comparisons were Holm-corrected. Because at most six complete dog-by-protocol blocks are available, Kendall W and per-arm means are reported alongside every p-value rather than p alone. For a Friedman test, W = chi-square / [n(k-1)] is a deterministic transform of the statistic, shown as standardised concordance rather than as independent evidence. With six blocks, the asymptotic chi-square p can be unreliable, so the Friedman p-values were confirmed by a within-block permutation null (20,000 permutations): global p = 0.31, ventilation p = 0.0008, raw arterial carbon dioxide p = 0.001, and raw heart rate p = 0.0001, all consistent with the asymptotic values. The four-panel re-analysis in Supplementary Table S7 used 10,000 within-block permutations and reproduces the full-panel value (p = 0.325).

**S4. Oxygen-band and band-width sensitivity**

Two sensitivity analyses test whether the headline conclusions depend on modelling choices. First, the oxygen-tension variables (PaO_2_, SvO_2_, PvO_2_), whose room-air bands are exceeded under near-pure inspired oxygen, were removed one at a time and together, and the between-protocol comparison was repeated (Supplementary Table S4, Supplementary Figure S10). Second, every band was scaled by k from 0.75 to 1.50 and the global and ventilation-subsystem comparisons were repeated (Supplementary Table S5). On the full variable panel, the between-protocol comparison remained non-significant and the ventilation-subsystem difference held throughout. This robustness concerns the band specification only, because the same comparison reverses with the variable panel on identical sessions (Supplementary Table S7). A third analysis re-specified the bands to anaesthesia-appropriate ranges and re-examined the variance partition without the oxygen-tension variables (Supplementary Results).

**S5. Ventilation mode**

The between-protocol difference in the recorded arterial carbon dioxide is most readily explained if carbon dioxide was not actively clamped to a fixed target, consistent with spontaneous or non-carbon-dioxide-targeted ventilation. The available data do not record the ventilation mode, so the mechanistic interpretation is inferred rather than stated. The main text accordingly does not read this difference as an effect of the protocols.

**S6. Candidate minimal panel for prospective validation**

A prospective evaluation of the score does not need every variable scored here. The importance map (Supplementary Figure S9) shows that severity was carried by a small set: mean arterial pressure and core temperature on the low side, and arterial and end-tidal carbon dioxide on the high side. Several scored variables never left their band and contributed nothing (pulmonary artery and wedge pressure), and others were redundant with mean arterial pressure (systolic and diastolic pressures) or negligible (central venous pressure). Heart rate is physiologically central but remained within the selected band, resulting in a negligible contribution to the score despite a large pharmacologic change, and the oxygen-tension variables breached high only because room-air bands were applied under therapeutic oxygen.

From this, Supplementary Table S6 gives a tiered candidate panel for prospective validation. The list is descriptive rather than recommended, recording which channels carried out-of-band weight in seven laboratory Beagles from a single research group, and it was not tested against any outcome. Tier 1 is a minimal, continuously and non-invasively available panel that reproduced the arc and the two-sided signature: mean arterial pressure, core temperature, end-tidal carbon dioxide, heart rate, and oxygen saturation. Oxygen saturation is scored as a one-sided floor rather than a corridor, because it sits at its ceiling under oxygen. Tier 2 adds variables that are informative only with corrected, state-aware bands or with invasive access (arterial oxygen and carbon dioxide tension, mixed-venous saturation). Tier 3 lists variables to drop or downweight because they contributed nothing or duplicated Tier 1.

The bands suggested in Table S6 are anaesthesia-appropriate directions rather than validated cut-points. They widen the awake bands towards the values expected under general anaesthesia, a lower mean-arterial-pressure threshold, a mild-hypothermia-tolerant temperature band, permissive hypercapnia, and an inspired-oxygen-indexed oxygen band, and each still needs a citable clinical source before use, as for the bands in Supplementary Table S1. Such an evaluation would also have to report depth (top three) and breadth alongside the root-mean-square score, because the root-mean-square dilutes as the number of available channels changes, which it will in a live setting. Two of the Tier 1 channels, end-tidal carbon dioxide and oxygen saturation, were recorded only after induction in these data, so the panel could not be scored across the whole anaesthetic course here, and prospective testing against an external anchor would be needed before any such panel is put forward for clinical monitoring.

**Supplementary Results**

Subsystem comparisons (Supplementary Table S2). The global test on the full panel returned Friedman p = 0.284 (W = 0.21), a verdict that is not stable across variable panels (Supplementary Table S7). The ventilation subsystem differed (p = 0.005, q = 0.03, W = 0.72) and was the only subsystem to reach significance after correction; temperature (p = 0.037) and the low-side panel (p = 0.050) leaned towards placebo but did not survive correction. The placebo lean is temperature-driven, since mean arterial pressure severity was flat across protocols. As stated in the main text, these subsystem results are reported as a property of the score under each variable subset rather than as evidence about the protocols.

Raw-variable confirmation (Supplementary Table S3). On raw maintenance values, arterial and end-tidal carbon dioxide differed between protocols (p = 0.007 and p = 0.009, methadone arms higher). Heart rate differed strongly (p = 0.002, W = 0.86) but rermained within the selected 50 to 118 beats per minute band, resulting in a negligible heart-rate contribution to the corridor score. Mean arterial pressure did not differ (p = 0.13 and p = 0.53), consistent with the cardiovascular results reported in the source study, and this analysis neither adds to nor qualifies them.

Awake and premedication window. The awake-window comparison returned Friedman p = 0.204 on the full panel, and it carries the same panel dependence as the maintenance comparison, so it is not read as evidence about tasipimidine sedation (main text, Figure 3D). Within this window, heart rate was already lower in the methadone arms (p = 0.014, uncorrected and exploratory; the Holm-corrected placebo-versus-arm tests did not survive), an early pharmacologic signal that the corridor did not score because it stayed in band.

Per-timepoint analysis (Supplementary Figure S12). No single timepoint differed between protocols after Benjamini-Hochberg correction; the smallest raw p was at M1 (0.025).

Clinical importance map (Supplementary Figure S9). The arterial carbon dioxide weight rose from 0.01 in placebo to 0.33 in the methadone-plus-dexmedetomidine arm, while the placebo arm carried the largest core-temperature (hypothermia) weight, the row that drives its low-side lean; the mean-arterial-pressure weights did not single out placebo.

Two-sidedness index (Supplementary Figure S11). Across maintenance samples, the fraction with a simultaneous low-side and high-side band breach ranged from 39% (dog 5) to 79% (dog 6), confirming that opposite departures co-occur within the same animal.

Band re-specification sensitivity (Supplementary Methods S4). The headline magnitudes were re-tested against an illustrative re-specification of the bands towards anaesthesia-appropriate ranges (mean arterial pressure 60 to 120, core temperature 37.0 to 39.2, heart rate 50 to 140, arterial and end-tidal carbon dioxide 35 to 55 mmHg; oxygen-tension variables dropped). Two results are band-robust, and two are band-dependent. Robust: on the full variable panel the between-protocol comparison stayed non-significant under every re-banding (Friedman p = 0.15 to 0.28), though this robustness concerns the band specification only, and the same comparison reverses with the variable panel (Supplementary Table S7), and the raw between-protocol carbon dioxide and heart rate differences (Table S3) do not depend on any band. Band-dependent: the phase share of the variance partition fell from 64% to about 20% once the oxygen-tension variables were removed and to about 1% under anaesthesia bands, with the within-session residual then dominating the partition, and the maintenance-to-awake severity ratio fell from 2.7 to 1.2, so the induction arc keeps its direction but loses most of its magnitude; and the ventilation corridor-severity difference weakened from p = 0.005 to a marginal p = 0.05 for the two-variable panel (p = 0.16 for arterial carbon dioxide alone) under the permissive 35 to 55 mmHg band, although the raw carbon dioxide difference is unchanged. The two-sided co-occurrence of low- and high-side breaches likewise fell from 54% of maintenance samples under the awake bands to 7% under anaesthesia bands. The corridor's structural addition over a one-sided score, the high-side carbon dioxide excursion present in about half of maintenance samples, is a matter of direction and does not depend on the band magnitude.

Panel dependence of the between-protocol comparison (Supplementary Table S7). Three of the 19 scored channels are recorded almost only under anaesthesia (respiratory rate, pulse oximetry and end-tidal carbon dioxide), so the score was recomputed from scratch on the fixed 16-variable phase-stable panel, on that panel without the four oxygen-tension channels (12 variables), and on a reduced panel carrying one channel per physiological system (7 variables), with the per-variable reference maxima rebuilt within each panel. The recorded course survives each recomputation, with the maintenance mean above the awake mean in 27, 25 and 27 of 27 sessions, and the awake-to-maintenance step moving from +0.202 on the full panel to +0.225, +0.120 and +0.308, so the size of the step is a property of the panel. The between-protocol verdict is not stable across the same four panels. The blocked Friedman test returns p = 0.284 on the full panel, p = 0.024 on the phase-stable panel, p = 0.334 without the oxygen tensions, and p = 0.012 on the one-channel-per-system panel, each reproduced by 10,000 within-block permutations (p = 0.325, 0.021, 0.372 and 0.005), on identical sessions and measurements. The full-panel and reduced-panel scores nevertheless track one another closely across all timepoints (Spearman ρ = 0.93). As reported in the main text, the between-protocol comparison is therefore withdrawn as evidence about the protocols, and the panel dependence is itself reported as a result about the score.

**Supplementary Tables**

**Supplementary Table S1.** Reference bands used for the corridor fold. Bands are either mean ± 2 SD from reference data or published clinical ranges. These are reference ranges rather than validated cut-points, and conclusions are checked for robustness to band width and re-specification (Supplementary Methods S4). The oxygen-tension bands (PaO_2_, SvO_2_, PvO_2_) are room-air ranges over-applied under therapeutic oxygen and breach high, as discussed; the saturation bands (SaO_2_, SpO_2_) cap at 100% and stay inside their band, so they add no false over-band severity but cannot resolve high oxygen tension.

| **Variable** | **Normal band** |
| --- | --- |
| Rectal temperature (°C) | 38.3 – 39.2 |
| Heart rate (bpm) | 50 – 118 |
| Respiratory rate (/min) | 8 – 20 |
| SpO_2_ (%) | 95 – 100 |
| etCO_2_ (mmHg) | 35 – 45 |
| SAP, NIBP (mmHg) | 84 – 152 |
| MAP, NIBP (mmHg) | 80 – 100 |
| DAP, NIBP (mmHg) | 40 – 92 |
| SAP, IBP (mmHg) | 84 – 152 |
| MAP, IBP (mmHg) | 80 – 100 |
| DAP, IBP (mmHg) | 40 – 92 |
| PaCO_2_ (mmHg) | 35 – 45 |
| PaO_2_ (mmHg) | 70 – 110 |
| Core temperature (°C) | 38.3 – 39.2 |
| PAP, mean (mmHg) | 5 – 25 |
| PAWP (mmHg) | 2 – 16 |
| CVP (mmHg) | 3 – 7 |
| SaO_2_ (%) | 95 – 100 |
| PvO_2_ (mmHg) | 30 – 50 |
| SvO_2_ (%) | 60 – 80 |

**Supplementary Table S2.** Between-protocol severity by clinical subsystem (Study 2 maintenance, Friedman blocked by dog, n = 6 complete blocks). q is the Benjamini-Hochberg value across the six panels. These subsystem verdicts are an instance of the panel dependence reported in the main text and are not read as evidence about the protocols.

| **Subsystem** | **Vars** | **PP** | **TP** | **TMP** | **TMPD** | **χ²** | **p** | **q** | **W** |
| --- | --- | --- | --- | --- | --- | --- | --- | --- | --- |
| Global | 19 | 0.357 | 0.302 | 0.322 | 0.313 | 3.8 | 0.284 | 0.284 | 0.21 |
| Cardiovascular | 10 | 0.269 | 0.221 | 0.249 | 0.225 | 5.0 | 0.172 | 0.206 | 0.28 |
| Blood pressure | 6 | 0.275 | 0.249 | 0.288 | 0.23 | 5.4 | 0.145 | 0.206 | 0.3 |
| Ventilation (CO_2_) | 2 | 0.055 | 0.021 | 0.224 | 0.352 | 13.0 | 0.005 | 0.028 | 0.72 |
| Temperature | 1 | 0.425 | 0.275 | 0.192 | 0.285 | 8.49 | 0.037 | 0.101 | 0.47 |
| Low-side | 3 | 0.465 | 0.397 | 0.41 | 0.361 | 7.8 | 0.05 | 0.101 | 0.43 |

**Supplementary Table S3.** Raw-variable between-protocol check (Study 2 maintenance, Friedman blocked by dog). Means are in the variable's own units. The ventilation variables differ (methadone arms higher); heart rate differs strongly but remains within the selected band; mean arterial pressure and SpO_2_ do not differ. Because the ventilation mode and the intra-anaesthetic management were not recorded, these raw differences are not attributed to the protocols.

| **Variable** | **n** | **PP** | **TP** | **TMP** | **TMPD** | **χ²** | **p** | **W** |
| --- | --- | --- | --- | --- | --- | --- | --- | --- |
| PaCO_2_ | 5 | 39.5 | 40.9 | 51.4 | 51.9 | 12.12 | 0.007 | 0.81 |
| etCO_2_ | 6 | 42.4 | 40.8 | 47.6 | 52.3 | 11.6 | 0.009 | 0.64 |
| Heart rate | 6 | 113.0 | 92.0 | 79.5 | 67.9 | 15.4 | 0.002 | 0.86 |
| MAP (IBP) | 5 | 73.6 | 71.7 | 65.6 | 73.0 | 5.57 | 0.134 | 0.37 |
| MAP (NIBP) | 6 | 62.0 | 61.4 | 60.8 | 63.4 | 2.2 | 0.532 | 0.12 |
| Core temperature | 6 | 37.3 | 37.6 | 37.9 | 37.7 | 9.0 | 0.029 | 0.5 |
| SpO_2_ | 6 | 99.8 | 99.6 | 99.8 | 99.2 | 4.02 | 0.26 | 0.22 |

**Supplementary Table S4.** Oxygen-band sensitivity (Study 2 maintenance). Removing the oxygen-tension variables lowers the severity level by about 30% but leaves the between-protocol comparison non-significant throughout, so the full-panel verdict is not produced by the oxygen artefact. The 16-variable row here (the full panel without PaO_2_, SvO_2_ and PvO_2_) is not the 16-variable phase-stable panel of Supplementary Table S7, which drops the three channels recorded only under anaesthesia instead and returns p = 0.024. Arm% is the between-protocol share of variance.

| **Panel** | **Vars** | **Median** | **PP** | **TP** | **TMP** | **TMPD** | **p** | **W** | **Arm%** |
| --- | --- | --- | --- | --- | --- | --- | --- | --- | --- |
| Full panel | 19 | 0.31 | 0.357 | 0.302 | 0.322 | 0.313 | 0.284 | 0.21 | 1.9% |
| Drop PaO_2_ | 18 | 0.24 | 0.305 | 0.223 | 0.253 | 0.252 | 0.145 | 0.3 | 5.2% |
| Drop SvO_2_ | 18 | 0.292 | 0.316 | 0.286 | 0.318 | 0.311 | 0.204 | 0.26 | 0.4% |
| Drop PaO_2_+SvO_2_ | 17 | 0.212 | 0.248 | 0.188 | 0.239 | 0.245 | 0.284 | 0.21 | 3.3% |
| Drop all O_2_ | 16 | 0.217 | 0.257 | 0.195 | 0.246 | 0.251 | 0.284 | 0.21 | 3.4% |

**Supplementary Table S5.** Band-width (k) sensitivity (Study 2 maintenance. On the full variable panel, the global comparison stays non-significant, and the ventilation-subsystem difference stays significant as every band is scaled from 0.75 to 1.50 about its centre. The dependence of the same comparison on the variable panel is given in Supplementary Table S7.

| **k** | **Global p** | **Global W** | **Global median** | **Ventilation p** | **Ventilation W** |
| --- | --- | --- | --- | --- | --- |
| 0.75 | 0.284 | 0.21 | 0.329 | 0.003 | 0.77 |
| 1.0 | 0.284 | 0.21 | 0.31 | 0.005 | 0.72 |
| 1.25 | 0.172 | 0.28 | 0.294 | 0.005 | 0.72 |
| 1.5 | 0.284 | 0.21 | 0.277 | 0.005 | 0.72 |

**Supplementary Table S6.** Candidate minimal panel for a prospective evaluation of the corridor score, tiered by how much each variable contributes to the score here and by how readily it is measured. Suggested bands are anaesthesia-appropriate directions (they widen the awake bands of Table S1 towards values expected under general anaesthesia), not validated cut-points, and each still needs a citable source. SpO_2_ is scored as a one-sided floor, not a corridor, because it sits at its ceiling under therapeutic oxygen. The list is descriptive rather than recommended. It records which channels carried out-of-band weight in seven laboratory Beagles from a single research group, it was not tested against any outcome, and its composition is band-dependent, since the largest maintenance contributor in these data was arterial oxygen tension scored against a room-air band. A panel of this kind cannot be assumed to inherit the behaviour reported here, because the panels examined did not behave alike (Supplementary Table S7).

| **Variable** | **Tier** | **Role here** | **How to score** | **Suggested band** |
| --- | --- | --- | --- | --- |
| MAP (NIBP or IBP) | 1 | dominant low-side driver | corridor (two-sided) | lower ~60–65 mmHg |
| Core temperature | 1 | hypothermia driver | corridor | ~37.0–39.2 °C |
| etCO_2_ | 1 | carried the ventilation-subsystem difference | corridor | ~35–55 mmHg (permissive) |
| Heart rate | 1 | central but stayed in band | corridor (wide) | ~50–140 bpm |
| SpO_2_ | 1 | universal, non-invasive | one-sided floor | penalise below ~95% |
| PaO_2_ | 2 | oxygenation; artefact under fixed band | corridor, FiO_2_-indexed only | index to inspired fraction |
| PaCO_2_ | 2 | arterial confirmation of etCO_2_ | corridor | ~35–55 mmHg |
| SvO_2_ / PvO_2_ | 2 | extraction; needs PA catheter | corridor, state-aware band | research / ICU |
| PAP, wedge pressure | 3 | never left band (scored 0) | drop unless indicated | — |
| Systolic / diastolic pressure | 3 | redundant with MAP | drop or downweight | — |
| Central venous pressure | 3 | negligible contribution | drop | — |
| Cardiac output, PvCO_2_ | 3 | no defensible band | exclude | — |

**Supplementary Table S7.** Panel dependence of the recomputed corridor score (Study 2). Each panel is scored from scratch with the per-variable reference maxima rebuilt within it. Phase means are across the 27 sessions, the step is maintenance minus awake, and the between-protocol test is the blocked Friedman test on one maintenance mean per dog and protocol (six complete blocks), with the permutation p from 10,000 within-block re-orderings of the protocol labels inside each dog. The verdict reverses across defensible panels of the same sessions, so the comparison is not read as evidence about the protocols (main text, Figure 3E).

| **Panel** | **Vars** | **Awake** | **Maint.** | **Recov.** | **Step** | **Maint > awake** | **Friedman p** | **Perm. p** | **W** |
| --- | --- | --- | --- | --- | --- | --- | --- | --- | --- |
| Full | 19 | 0.121 | 0.323 | 0.167 | +0.202 | 27/27 | 0.284 | 0.325 | 0.21 |
| Phase-stable | 16 | 0.122 | 0.346 | 0.168 | +0.225 | 27/27 | 0.024 | 0.021 | 0.52 |
| Phase-stable without O_2_ tensions | 12 | 0.139 | 0.258 | 0.188 | +0.120 | 25/27 | 0.334 | 0.372 | 0.19 |
| One channel per system | 7 | 0.127 | 0.435 | 0.179 | +0.308 | 27/27 | 0.012 | 0.005 | 0.61 |

**Supplementary Figures**

**Supplementary Figure S1.** The same illustrative physiological trajectory scored under three severity rules. (A) One-sided RELSA penalises only declines below baseline, so a high excursion is invisible. (B) The PVS point reference penalises any deviation from a single normative value, with sub-reference values mirrored above. (C) The corridor used here penalises only excursions outside a normal band [lower, upper], with e = max(0, lower − x, x − upper) and pseudo-value 100 − e, and is zero inside the band. The only change from PVS is panel B to C, a tolerance band in place of a point reference.

**Supplementary Figure S2.** Association with arterial acid-base (Study 2). (A) top-three severity, (B) arterial pH, (C) base excess across the M0 to M9 course, with phase shading and the documented induction at M3 (dashed). The calculated corridor score peaks where pH and base excess reach their nadir. Within-session correlations are severity versus pH, r = −0.70, and severity versus base excess, r = −0.56; neither is in the score. The partial correlation between base excess and arterial carbon dioxide is −0.20, so much of the acidosis is respiratory, with only a weak residual metabolic component.

**Supplementary Figure S3.** Aggregation robustness (Study 2). The same per-variable corridor weights aggregated four ways: the canonical root-mean-square score, the top-three mean (depth), the maximum (worst single system), and the fraction of measured systems out of band (breadth). Under the selected bands, the temporal pattern of the calculated score across induction, maintenance, and recovery is retained under all four aggregation rules, with the rise occurring at the documented propofol induction (dashed M3). Mean and standard error across 27 sessions, with an independent y-scale per panel.

**Supplementary Figure S4.** Corridor severity against the published dose-sparing gradient (Study 2). (A) Propofol induction dose falls from 0.71 to 0.29 mL/kg across the four protocols (Pearson r = −0.81 with protocol order), the published dose-sparing effect of Kästner et al. (2024a). (B) Session-mean corridor severity by protocol under four aggregations. The session-mean score did not rise with the sparing gradient under any aggregation (Spearman ρ = −0.35, −0.30, −0.05, −0.13 for the canonical score, top-three depth, worst single system, and breadth); the Spearman values are computed across the four protocol means and are descriptive. Protocol order is PP, TP, TMP, TMPD. This is not read as evidence about the protocols, because the between-protocol verdict of the score depends on the variable panel (main text, Figure 3E, and Supplementary Table S7).

**Supplementary Figure S5.** The bidirectional pattern reproduces across both datasets. (A) Direction-resolved corridor contributions for the variables shared by both studies (Study 1 isoflurane sparing and Study 2 cardiovascular). The low-side pattern, mean arterial pressure and temperature breaching low (hypotension and hypothermia), reproduces in both; arterial oxygen tension breaches high in Study 2, with only a small Study-1 bar because it was sparsely sampled there (n = 46). (B) Study 1 inspired-oxygen fraction (n = 803): the dogs are ventilated on near-pure oxygen (median 97%, against room air 21%), the inspired-oxygen context for the high arterial oxygen tension observed in Study 2.

**Supplementary Figure S6.** Per-dog severity small multiples (Study 2), one panel per dog, ordered by peak severity. Grey lines are individual sessions, and the coloured line is the dog’s mean. Phase shading and the dashed propofol induction (M3) are as in the main arc. These panels are descriptive; the between-protocol comparison is shown in the main Figure 3, where its dependence on the variable panel is reported.

**Supplementary Figure S7.** Study 1 trajectories and corridor drivers (isoflurane-sparing dataset). (A) Per-dog severity across the four measurement days. Isoflurane was titrated up and down against a nociceptive stimulus rather than held at a fixed level, and sampling covered the maintenance period rather than the induction transition, so no induction arc appears. (B) Mean corridor weights: the low-side cardiovascular and thermal pattern (mean arterial pressure, temperature) mirrors Study 2 (0 = always in band, 1 = worst observed here).

**Supplementary Figure S8.** Why the corridor scores band-crossing, not in-band shifts (Study 2 maintenance, per dog-by-protocol mean, band shaded). (A) Heart rate shows a large between-protocol shift (113 to 68 bpm, p = 0.002) that remains within the 50 to 118 beats per minute band and therefore results in a negligible contribution to the calculated corridor score. (B) Arterial carbon dioxide rises above the 35 to 45 band in the methadone arms (p = 0.007). (C) Mean arterial pressure is below its selected band and flat across protocols (p = 0.13). For the calculation, the selected band therefore determines whether a change contributes to the corridor score.

**Supplementary Figure S9.** Corridor contribution map (Study 2 maintenance): mean corridor weight per variable per protocol (0 = always in band, 1 = worst observed). Rows are ordered by overall weight. The arterial carbon dioxide gradient (placebo 0.01 to methadone-plus-dexmedetomidine 0.33) and the low-side hypotension and hypothermia rows are the dominant score contributions. The oxygen-tension rows (PaO_2_, SvO_2_, PvO_2_, marked †) reflect a room-air band applied under near-pure oxygen and are a band artefact, not pathology.

**Supplementary Figure S10.** Oxygen-band sensitivity (Study 2 maintenance). (A) Mean severity per protocol as the oxygen-tension variables are removed: the four protocols stay together and the level drops about 30%. (B) The between-protocol Friedman p stays above 0.05 throughout, so the full-panel verdict is not produced by the oxygen artefact. The dependence of the same comparison on the variable panel is shown in Supplementary Table S7.

**Supplementary Figure S11.** Per-dog two-sidedness exemplars, selected objectively as the strongest (dog 6, 79%) and weakest (dog 5, 39%) by the fraction of maintenance samples with a simultaneous low-side and high-side band breach. Each panel shows the raw course of two low-side variables (mean arterial pressure, core temperature) and one high-side variable (arterial carbon dioxide), one line per protocol, with the corridor band shaded and the maintenance window in grey. The strong dog breaches low and high at the same time.

**Supplementary Figure S12.** Per-timepoint between-protocol comparison (Study 2). (A) Mean severity per protocol across M0 to M9; the four protocols track together. (B) The per-timepoint Friedman p with a 0.05 reference. No timepoint survives Benjamini-Hochberg correction; the smallest raw p is at M1 (0.025), so there is no robust earliest timepoint of difference.
